# Combined Impact of Creatine, Caffeine, and Variable Resistance on Repeated Sprint Ability in Young Soccer Players

**DOI:** 10.3390/nu16152437

**Published:** 2024-07-26

**Authors:** Álvaro Huerta Ojeda, Carlos Jorquera-Aguilera

**Affiliations:** 1 Núcleo de Investigación en Salud, Actividad Física y Deporte ISAFYD, Universidad de Las Américas, Viña del Mar 2531098, Chile; 2 Facultad de Ciencias, Escuela de Nutrición y Dietética, Universidad Mayor, Santiago 8580745, Chile; carlos.jorquera@mayor.cl

**Keywords:** ergogenic aid, post-activation performance enhancement, athletic performance, soccer

## Abstract

There is evidence that both intra-serial variable resistance (I-sVR), as pre-activation within the post-activation performance enhancement cycle (PAPE), and creatine and caffeine supplementation increase athletic performance in isolation. However, the effect of the three conditioning factors on 30 m repeated sprint ability (RSA) performance in young soccer players is unknown. This study determined the summative and isolation effect of ergogenic aids and pre-activation in half-back squats (HBSs) with I-sVR on performance in an RSA test in young soccer players. Twenty-eight young soccer players were randomly assigned to either EG_1_ (*n* = 7, creatine + caffeine + I-sVR), EG_2_ (*n* = 7, creatine + placebo_2_ + I-sVR), EG_3_ (*n* = 7, placebo_1_ + caffeine + I-sVR), or EG_4_ (*n* = 7, placebo_1_ + placebo_2_ + I-sVR), using a factorial, four-group-matched, double-blind, placebo-controlled design. Creatine supplementation included 0.3 g/kg/day for 14 days, caffeine supplementation included 0.3 mg/kg per day, and pre-activation in HBS with I-sVR (1 × 5 at 30% 1RM [1.0–1.1 m/s] + 1 × 4 at 60% 1RM [0.6–0.7 m/s]). The RSA test and HBS outcomes were evaluated. Three-way ANOVA showed non-significant differences for the RSA test and HBS outcomes (*p* > 0.05). At the end of this study, it was found that the three ergogenic aids, together, do not generate a summative effect on the physical performance of young soccer players. However, it is important to analyze individual responses to these specific protocols.

## 1. Introduction

Modern soccer is characterized by intermittent high-intensity efforts combined with moderate-to-low-intensity efforts, periods of limited activity, and stationary rests [1]. During the development of high-intensity intermittent efforts, muscular strength, and power, along with speed and acceleration, are conditioning elements for sports performance [2]. In this context, post-activation performance enhancement (PAPE) is a method that has been used to increase muscle power transiently [3,4]. PAPE is based on the phosphorylation of myosin light chains, increasing the recruitment of higher-order motor units and changes in the tendon pennation angle [5,6]. In this scenario, different exercises have been used [7], highlighting the half-back squat (HBS) as one of the most used exercises for this purpose [8]. Indeed, there is evidence that HBS performed within an intra-series variable resistance (I-sVR), as a pre-activation to trigger PAPE, generates increases in muscle power, as evidenced by performance over 30 m [9,10]. Physiologically, I-sVR increases the excitability of motor neurons [11], causing increased myosin light chain phosphorylation, ATPasa activity, muscle fiber contractile capacity, and motor unit recruitment, mainly of type II muscle fibers [5,6]. For more details on the features of I-sVR, go to Section 2.8. Despite the evidence showing a positive effect of PAPE on physical performance [8], to the best of our knowledge, no information determines the effect of pre-activation in a HBS with I-sVR to trigger PAPE on performance in 30 m repeated sprint ability (RSA) tests in young soccer players.

Within ergogenic aids, nutritional supplements are essential to improve physical performance [12]. Indeed, creatine monohydrate is one of the substances most used by athletes [13,14], showing increases in muscle strength, power, sprints, and exercises, requiring short bursts of energy (anaerobic metabolism) [15,16,17,18]. A preliminary study concluded that a dose of orally administered creatine (0.3 g/kg/day) for 14 days generated physical performance gains in young soccer players [19]. From a physiological perspective, this effect is generated because creatine and phosphagen catalyzed by the enzyme creatine kinase (CK) play a fundamental role in the energy supply in the anaerobic alactic metabolism. This synthesizes ATP from phosphocreatine (PCr) and adenosine diphosphate (ADP) [20]. In addition, other research has shown favorable results on using creatine in the anaerobic metabolism of professional and youth soccer players [21,22]. These conclusive results allow the safe administration of this nutritional supplement. However, the summative effect of a dose of orally administered creatine and pre-activation in HBSs with I-sVR on 30 m RSA performance in young soccer players is unknown.

Another nutritional supplement widely used to increase physical performance is caffeine, synthesized as caffeine anhydrous [23]. This ergogenic aid has been used in both aerobic endurance sports and strength sports, showing improvements in muscular endurance, speed of movement, and muscular strength [24]. Likewise, using caffeine has demonstrated improvements in post-exercise recovery [25]. Concerning the dose and age of caffeine consumption, scientific evidence has shown that doses between 3 and 6 mg/kg/day of caffeine can increase physical performance in trained athletes [25,26,27]. Also, evidence shows that acute ingestion of caffeine with doses of 0.9–3.2 mg/kg/day [28], 3–5 mg/kg/day [29], or 1–3 mg/kg/day [26] increases physical performance without the presence of side effects in physically trained children and adolescents. However, if participants have no experience with caffeine intake as an ergogenic aid and if individual physiological responses to this supplement are unknown, it is advisable to start with the lowest doses that have scientific evidence [26,29]. Consequently, an acute supplementation of 3 mg/kg/day of caffeine ingested one hour before exercise or training should increase physical performance in trained athletes between 15 and 22 years of age [24]. However, the acute effect of orally administered caffeine, along with pre-activation in HBSs with I-sVR, on 30 m RSA performance in young soccer players is unknown. There is also no evidence of the summative effect of a dose of creatine administered orally for 14 days, a single dose of caffeine, and pre-activation in HBSs with I-sVR on 30 m RSA performance in young soccer players.

Although there is evidence that pre-activation in HBSs with I-sVR triggers PAPE and that both creatine and caffeine increase muscles’ contractile capacity, there is no certainty about the summative effect of the three conditions on 30 m RSA performance in young soccer players. For this reason, the main objective of this research was to determine the summative effect of ergogenic aids and pre-activation in HBSs with I-sVR on 30 m RSA performance in young soccer players. The secondary objective was to determine the isolated effect of the three treatments and the velocity of execution and power achieved in HBSs. The sum effect of creatine supplementation + caffeine + pre-activation in HBSs with I-sVR was hypothesized to be greater than that of each isolated treatment.

## 2. Materials and Methods

### 2.1. Design

**Figure 1:**
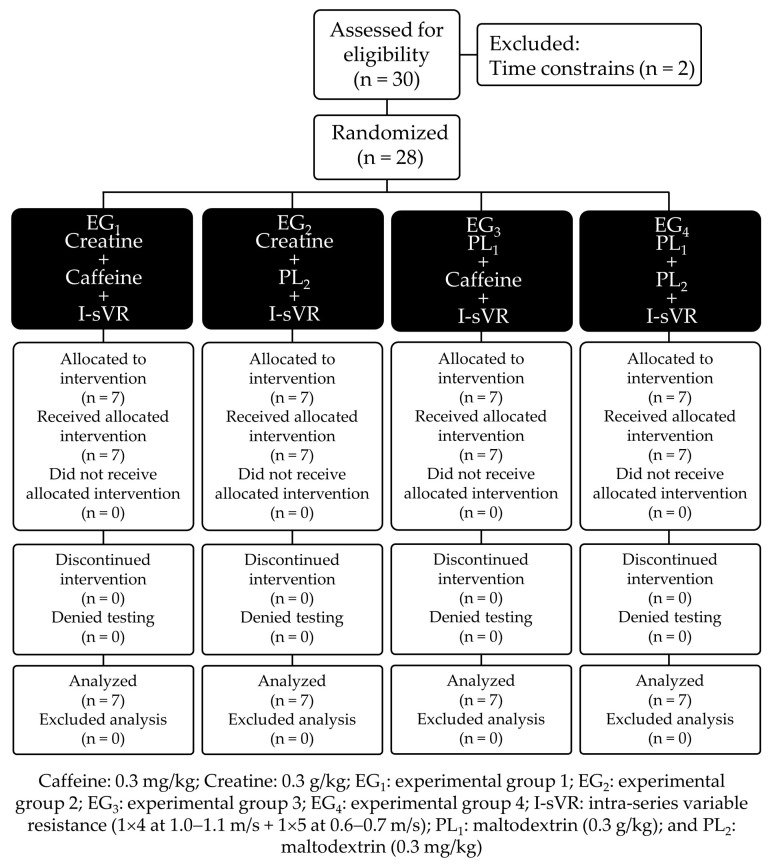
Research design.

**Figure 2:**
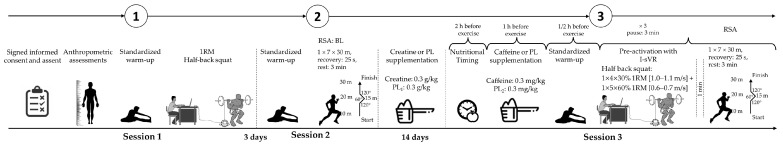
Study process overview.

The present study, as well as a study entitled “Effects of a Low Dose of Orally Administered Creatine Monohydrate on Post-Fatigue Muscle Power in Young Soccer Players” [19], was part of a macro project to determine the effect of ergogenic aids on physical performance in young soccer players. Consequently, some procedures and evaluations are similar between the two. The present study’s design was factorial, double-blind, and placebo-controlled [30]. The intervention considered 28 participants distributed in four experimental groups (EG_1_, *n* = 7; EG_2_, *n* = 7; EG_3_, *n* = 7; and EG_4_, *n* = 7). The first part of this study considered chronic creatine supplementation for 14 with a dose equivalent to 0.3 g/kg administered orally to EG_1_ and EG_2_ [18] or a placebo (PL) of maltodextrin equivalent to 0.3 g/kg for EG_3_ and EG_4_. The second part of this study included supplementation with a single dose of caffeine equivalent to 0.3 mg/kg for EG_1_ and EG_3_ [18] or a PL of maltodextrin equivalent to 0.3 mg/kg for EG_2_ and EG_4_. Sixty minutes after caffeine intake, to trigger PAPE, participants performed a pre-activation in HBS with I-sVR (Figure 1). For more details on the features of I-sVR, go to Section 2.8. Before and after the intervention, to evaluate the isolated and summative effect of VR and ergogenic aids, the young soccer players underwent an RSA test: 3 × 7 × 30 m, recovery 25 s, and rest 3 min (Figure 2).

### 2.2. Participants

Twenty-eight young soccer players from the Everton Club of Viña del Mar, Chile, volunteered to participate in this study. The sample was classified as “Trained” [31] and distributed by sample matching (EG_1_, *n* = 7; EG_2_, *n* = 7; EG_3_, *n* = 7; and EG_4_, *n* = 7). The inclusion criteria considered (a) being male, (b) being between 16 and 20 years old, (c) belonging to a youth division of a professional soccer club, and (d) having a minimum of two years of experience in HBS overload. On the other hand, the exclusion criteria included muscle injuries, tendon injuries, or fractures occurring in the last three months, which prevented the performance of the tests proposed in this study. Recruitment of participants and evaluations were performed between December 2023 and January 2024. Both the inclusion and exclusion criteria were described in Huerta Ojeda et al. [19]. Informed consent was provided in writing and signed before the start of the assessments. Similarly, all young soccer players, regardless of age, along with the guardians of minor participants, were fully informed of this study’s objectives before signing informed consent and assent forms. Participants under 18 years old signed informed assent forms, while their guardians and participants aged 18 and over signed informed consent forms. The study protocol, informed consent forms, and informed assent forms were approved by the Ethical-Scientific Committee of Universidad de Las Américas, Chile (registration number: CEC_FP_2023055). All study procedures were conducted under the Declaration of Helsinki (updated in 2013) and the ethical standards for exercise and sports [32].

### 2.3. Anthropometry

A digital scale (TANITA, model InnerScan BC-554^®^, Tokyo, Japan) was used to determine body weight (kg) and fat mass, while height (m) was measured with a stadiometer (SECA, model 700^®^, Hamburg, Germany). Weight and height measurements were performed in underwear. Body mass index (BMI) was calculated by dividing the body weight in kilograms (kg) by height in square meters (m^2^).

### 2.4. Warm-Up Protocol

The participants performed a standard 20 min warm-up consisting of 1 × 10 repetitions (reps) of plantar flexion and dorsiflexion of the ankles, 1 × 10 repetitions of flexion and extension of the knees, 1 × 10 repetitions of flexion and extension of the hips, and 1 × 10 repetitions of flexion, extension, adduction, and abduction of the shoulders. They then jogged for 6 min at an 8 km/h speed. They then added 2 × 10 s of leg, thigh, and hip muscle stretches. For the 1RM test on the HBS, they performed the following specific warm-ups: 1 × 6 × 20 kg, 1 × 4 × 25 kg, 1 × 4 × 30 kg, and 1 × 2 × 40 kg. For the RSA test, they performed the following specific warm-up: 10 × 30 m skipping, 3 × 10 m heel–glute exercise, four linear multiple jumps + 10 m running, and four lateral multiple jumps + 10 m running [19].

### 2.5. 1RM Test

The one repetition maximum (1RM) test in HBSs was incremental, starting with 20 kg (barbell only) until failure or until the program estimated the participant’s 1RM. All the participants were asked to lift the weight vertically as fast as possible for each load performed. In addition, during the test, the participants received verbal motivation from the research team [33]. A Chrono Jump^®^ linear encoder and the Chrono Jump version 1.4.6.0^®^ software (Barcelona, Spain) were used to evaluate the 1RM in HBSs [34]. During the evaluation of the 1RM, the progression of the loads (kg) and the corresponding execution velocities were recorded (m/s). Thus, the quantification of the load during pre-activation in HBSs with I-sVR was a function of the speed of movement (m/s) [35], using a linear encoder for this purpose [36].

### 2.6. RSA Test

The RSA set included seven repetitions of 30 m with three direction changes [37,38]. The first change of direction was performed at 10 m (120° to the right), the second at 15 m (60° to the left), and the third and last at 20 m (120° to the right) (Figure 2). All participants performed the RSA protocol on two occasions. The first time was before the supplementation protocols (creatine and caffeine), including one set of seven repetitions with 25 s passive recovery between repetitions. This RSA set was considered the baseline (BL). The second time was after the application of the creatine and caffeine supplementation protocols and after each set of pre-activation with I-sVR. This second time, three sets of RSAs were included. Each set of RSAs included seven 30 m repetitions with 25 s of passive recovery between repetitions. At the end of each set of RSA and before the next pre-activation with I-sVR, there was a 3 min rest (each set was performed after pre-activation with I-sVR). These RSA sets were considered to determine the isolated and summative effect of the creatine, caffeine, and I-sVR protocols. During the RSA evaluations, time was recorded using a Chrono Jump^®^ photocell and the Chrono Jump version 1.4.6.0^®^ software (Barcelona, Spain). A gantry was installed at the beginning (0 m) and another at the end of the run (30 m). The RSA test was performed outdoors on natural grass. The players wore soccer boots and received verbal support from the research team during the test [33]. For the present study, each set of RSAs was analyzed independently. At the same time, the statistical analysis included the best time (s), the mean time (s), the total time (s), the best speed (km/h), the mean speed (km/h), and the fatigue index (FI) (%). The FI was calculated using the following equation [39]:

$$Fatigue\;Index=\left(\frac{mean\;time\;of\;7\;rep-best\;time}{best\;time}\right)\times 100$$

### 2.7. Supplementation

#### 2.7.1. Creatine Supplementation

The EG_1_ and EG_2_ were supplemented with creatine for 14 days. The daily dose was equivalent to 0.3 g/kg/day [18,19]. Creatine was dissolved in 200 mL of water and ingested orally between 18:00 and 20:00 h [40]. The participants were asked to ingest creatine with a carbohydrate-rich meal to optimize creatine absorption [41]. EG_3_ and EG_4_ were supplemented with maltodextrin for 14 days (PL_1_). The daily dose was equivalent to 0.3 g/kg/day. All the young soccer players (EG_1_, EG_2_, EG_3_, and EG_4_) were asked to refrain from consuming caffeine, yerba mate, alcoholic beverages, carbonated beverages, isotonic beverages, energy drinks, protein shakes, supplements defined as activators, or any substance that increased their metabolism during the three weeks of the experiment. During the experiment, no specific diet was administered. However, the participants were asked to maintain the diet prescribed by the Everton club. These dietary guidelines were aligned with the nutritional needs corresponding to the energy demands of training and matches during this study.

#### 2.7.2. Caffeine Supplementation

The EG_1_ and EG_3_ were supplemented with caffeine. The single dose of caffeine was equivalent to 0.3 mg/kg [24]. Caffeine was dissolved in 200 mL of water and ingested orally one hour before exercise [25]. EG_2_ and EG_4_ were supplemented with maltodextrin (PL_2_). The dose was equivalent to 0.3 mg/kg. All young soccer players (EG_1_, EG_2_, EG_3_, and EG_4_) were asked to refrain from consuming caffeine, yerba mate, alcoholic beverages, carbonated beverages, isotonic beverages, energy drinks, protein shakes, supplements defined as activators, or any substance that increases the metabolism during the three weeks of the experiment. To avoid caloric deficit fatigue, all the participants received a pre-exercise snack [42,43,44]. The snack consisted of a carbohydrate load before caffeine supplementation. For this purpose, all the athletes were available to the investigators 2 h before the tests under fasting conditions. The nutritional timing consisted of 2 g of rapidly absorbed carbohydrate per kg body weight (2 g/kg) (Figure 2).

### 2.8. Pre-Activation in HBSs with I-sVR to Trigger PAPE

Muscle power can be transiently increased through a method called PAPE [3,4]. The description of PAPE establishes three phases: Phase 1 is the evaluation of an unpowered physical capacity or motor gesture; Phase 2 includes the application of a stimulus that triggers an increase in muscle power and/or movement speed, a phase which has also been described in the literature as pre-activation [45]; and Phase 3, corresponding to the re-evaluation of the physical capacity or motor gesture measured in Phase 1. In practical terms, the level of muscle power in Phase 3 of the PAPE cycle is expected to increase compared to Phase 1 [7]. For this study, the pre-activation phase consisted of one set of HBSs with I-sVR, based on the following execution velocity (m/s): 5 repetitions with a load equivalent to 1.0–1.1 m/s (30% 1RM) + 4 repetitions with a load equivalent to 0.6–0.7 m/s (60% 1RM) [7,8,9,10]. The pre-activation phase was before each set of RSAs. Therefore, three pre-activations in HBSs with I-sVR were performed during session 3. All the participants were asked to lift the weight vertically as fast as possible for each load. In addition, during the test, the participants received verbal encouragement from the research team [33]. A Chrono Jump^®^ linear encoder and the Chrono Jump version 1.4.6.0^®^ software (Barcelona, Spain) were used to evaluate the three HBS sets [34]. In the statistical analysis, the mean velocity (m/s) and the mean power (W) were the variables used to quantify the differences, isolated and summative, in creatine and caffeine supplementation and pre-activation with I-sVR.

### 2.9. Statistical Analysis

Descriptive data are presented as means and standard deviations (SDs). Considering that there were 28 participants, the normal distribution of the data was confirmed by the Shapiro–Wilk test (*p* > 0.05). For the comparison between groups before the application of the supplementation protocols and the I-sVR protocol, a one-way repeated measures analysis of variance (one-way ANOVA) was applied (*p* ˂ 0.05). Age and muscle strength (1RM) were used for sample matching. A three-way ANOVA (creatine vs. PL × caffeine vs. PL × time) was performed to assess the summative effect on RSA (best time, mean time, total time, best speed, mean speed, and fatigue index) and HBS (velocity and power) outcomes. The effect size (ES) was estimated by calculating the partial eta-squared (${ŋ}_{p}^{2}$): between 0.01 and 0.06, small, 0.07–0.14 medium, and ≥0.14 large effects. Additionally, one-way ANOVA was used to calculate the effect of each treatment. The latter analysis included the Bonferroni post hoc test. A significance level of *p* < 0.05 was accepted for all statistical comparisons. All statistical analyses were performed with the Prism version 10.2.0 for Windows^®^ software.

## 3. Results

At the time of the study, the four experimental groups showed non-significant differences in age, anthropometric characteristics, and basal physical performance (*p* > 0.05). The comparison between groups is shown in Table 1.

Variations were observed in both the RSA and HBS results between BL and post-intervention. Table 2 presents the mean values and SD of the RSA (best time, mean time, total time, best speed, mean speed, and fatigue index) and HBS (speed and power) results.

Three-way ANOVA showed non-significant changes in the seven comparisons performed for the RSA test results (*p* > 0.05). The same analysis showed non-significant changes in the time × creatine vs. PL and time × caffeine vs. PL for HBS outcomes (*p* > 0.05). The seven comparisons by outcome for the RSA and HBS test are presented in Table 3.

## 4. Discussion

The present study determined the summative effect of creatine supplementation, caffeine supplementation, and pre-activation in HBSs with I-sVR on 30 m RSA performance in young soccer players. The study results showed that the joint supplementation of creatine and caffeine, plus I-sVR, generated non-significant changes in the RSA test performance of young soccer players, rejecting the study hypothesis. When the treatments were analyzed in isolation (the secondary objective of the present study), all groups showed non-significant changes in the RSA test outcomes (*p* > 0.05) and HBS outcomes (*p* > 0.05). Nevertheless, EG_2_ showed non-significant increases in the RSA test performance in all three experimental series (S1, S2, and S3), EG_3_ showed non-significant increases in the RSA test performance in two of the three experimental series (S1 and S2), and EG_4_ showed non-significant increases in the RSA test performance in the first experimental series (S1). Finally, both EG_1_ (S1 and S2) and EG_3_ (S1, S2, and S3) showed non-significant increases in performance during pre-activation in HBS with I-sVR.

### 4.1. Creatine, Caffeine, and I-sVR on RSA Performance

As previously described, the joint use of creatine, caffeine, and pre-activation in HBSs with I-sVR did not synergistically affect RSA test performance in young soccer players (*p* > 0.05). However, the isolated analysis evidenced that these three ergogenic aids together non-significantly increased HBS performance (*p* > 0.05). Although our study hypothesis was rejected, the three ergogenic aids should have generated a synergistic effect on muscle power. This assumption was based on four antecedents: (a) the capacity of creatine to re-synthesize ATP in maximum-intensity efforts [46], (b) the capacity of caffeine to improve muscle performance [13], (c) the ability of the I-sVR to trigger PAPE [8], and (d) that the three ergogenic aids work through different metabolic pathways [47]. For example, creatine increases the intramuscular bioavailability of PCr [46,47], creating an environment conducive to the maintenance of cross-bridges and, thus, muscle power [48,49]. On the other hand, caffeine generates an antagonism of adenosine A1 and A2A receptors [25]. This antagonism of adenosine receptors causes an increase in neurotransmitter release and motor unit firing frequency, increasing muscle power [13]. There is also conclusive evidence that I-sVR increases muscle power through PAPE [9,10], mainly because I-sVR increases the excitability of motor neurons [11], increasing ATPasa activity mainly in fast-twitch fibers [5,6]. However, although current research suggests that acute caffeine supplementation does not alter the effect of chronic creatine [13], there is also evidence to suggest that combined creatine and caffeine supplementation eliminates the ergogenic effects of creatine during intense intermittent exercise, mainly by counteracting calcium (Ca^++^) clearance in the sarcoplasmic reticulum [47,50,51]. In this sense, Trexler et al. [52] concluded that a creatine load in combination with anhydrous caffeine does not improve sprint performance, showing a statistically significant deterioration of anaerobic performance in people with experience in endurance training [52]. Likewise, Mabrey et al. [53] evidenced that the co-ingestion of creatine and caffeine nitrate generates non-significant changes in the physical performance of people with experience in endurance training. Despite the similarity in the results, the present study pioneers exploring the summative effect of creatine and caffeine, plus a pre-activation in HBS with I-sVR, on physical performance in young soccer players. Eventually, the non-significant increase observed in HBS is generated by the location of the pre-activation phase within the methodological design (Figure 2) and the physical level of the young soccer players at the time of the study [31]. Apparently, because of the synergy of the ergogenic aids, the young players were only able to increase, non-significantly (*p* > 0.05), velocity and power in HBSs, without having the ability to maintain this increased performance during the RSA test, despite their “Trained” status [31]. Therefore, and despite the results, the synergistic effect of (a) chronic supplementation with a dose of creatine, (b) acute supplementation with a dose of caffeine, and (c) pre-activation in HBS with I-sVR to trigger PAPE in young soccer players requires further exploration, analyzing both the periods, times, and doses of intake and the loads to trigger PAPE. Finally, it is important to analyze individual responses to these specific protocols (see Supplementary Materials).

### 4.2. Creatine and I-sVR on RSA Performance

The isolated analysis of time × creatine vs. placebo showed non-significant changes in performance in the RSA test and HBS (*p* > 0.05). In this context, Yáñez-Silva et al. [54] observed an increase in the peak and average power in young soccer players after ingesting 0.3 g/kg/day of creatine for 14 days (same protocol used in the present study). However, despite the similarity of the intake protocol used in the study by Yáñez-Silva et al. [54] to the present study, in the former, a protocol in HBSs with I-sVR as a pre-activation to trigger PAPE was not included. Although our study hypothesis was rejected, the two ergogenic aids should have generated a synergistic effect on muscle power. To our knowledge, the I-sVR protocol applied after the 14 days of creatine supplementation and before the RSA test was expected to generate a synergistic effect between both ergogenic aids [7,8]. This is supported both by the increased intramuscular bioavailability of creatine [46] and the reduction in relaxation time [47,51], events which favor ATP re-synthesis and increased physical performance in young soccer players [17]. In this scenario, the supplementation protocol with creatine (0.3 g/kg/day creatine for 14 days) and the protocol in HBSs with I-sVR (1 × 5 at 1.0–1.1 m/s [30% 1RM] + 1 × 4 at 0.6–0.7 m/s [60% 1RM]), together, were shown to be efficient in increasing physical performance in young soccer players. Therefore, training processes should include both protocols to optimize physical performance [7,8,55].

### 4.3. Caffeine and I-sVR on RSA Performance

At the end of the intervention, non-significant changes were observed in the RSA and HBS test results (*p* > 0.05). Also, the isolated analysis of caffeine supplementation and pre-activation in HBSs with I-sVR showed non-significant increases in performance in the RSA test and HBSs (*p* > 0.05). Despite the non-significant differences, the results of these two ergogenic aids demonstrate synergy in the physical performance of young soccer players, improving the mean time, the total time, and the FI of the RSA test and the mean velocity and the power in HBSs. In this context, Ellis et al. [26] evidenced that 3 mg/kg of caffeine increases performance in explosive tests, while Guest et al. [24] concluded that the longest time for caffeine administration is 60 min before exercise (the same protocol used in the present study). However, the same authors mention that the optimal time for caffeine intake depends on the source of caffeine [24]. Despite the similarity of the protocols, Ellis et al. [26] and Guest et al. [24] did not include a protocol in HBSs with I-sVR as a pre-activation phase to trigger PAPE. From a physiological perspective, caffeine intake increases the release of neurotransmitters, increases the firing frequency of motor units [13], decreases extracellular potassium (K^+^), and increases excitation–contraction coupling [47]. These events favor the increase in muscle power. From a practical perspective, the non-significative changes observed in the EG_3_, specifically in the S1 and S2 in the RSA test and the S1, S2, and S3 in HBSs, could support the use of both ergogenic aids (caffeine 0.3 mg/kg and HBSs with I-sVR 1 × 5 at 1.0–1.1 m/s [30% 1RM] + 1 × 4 at 0.6–0.7 m/s [60% 1RM]), as they have been shown to be effective in increasing physical performance in young soccer players.

### 4.4. I-sVR on RSA Performance

At the end of the intervention, there were no significant changes in the RSA test results (*p* > 0.05). Also, the isolated analysis of pre-activation in HBSs with I-SVR showed non-significant increases in performance in the RSA test (*p* > 0.05). Despite the non-significant differences, the results demonstrate an increase in the physical performance of young soccer players, improving the mean time, the total time, and the mean speed of S1, as well as the FI of S1, S2, and S3 during the RSA test. The VR has shown good results in developing muscular strength and power [7,8], while its main characteristic is the intensity variation within the training load [56]. As described above, due to its action on the neuromuscular system, the use of RV as a pre-activation allows the triggering of PAPE, mainly of type II muscle fibers [5,6]. In previous studies, we tested the effect of pre-activation in HBSs with I-sVR (1 × 5 at 1.0–1.1 m/s [30% 1RM] + 1 × 4 at 0.6–0.7 m/s [60% 1RM]) on 30 m performance, showing increases in physical performance in both women [9] and men [10]. Therefore, this methodology has proven efficient in transiently increasing muscle power in trained individuals. However, it is essential to analyze the individual responses generated with this pre-activation methodology (see Supplementary Materials).

### 4.5. Limitations

During the development of this study, the intake of creatine and caffeine contained in the daily diet of the study participants was not calculated, nor was a calculation of the total daily caloric expenditure of the young soccer players. The participants performed the RSA test at the baseline, without pre-activation, in HBSs with I-sVR. After the supplementation protocols, they performed the RSA test with pre-activation in HBSs with I-sVR. Future studies should randomize participants using these protocols. The results reported in the present study represent young male soccer players. Therefore, young female soccer players should be evaluated to test the effects of these ergogenic aids. Finally, perhaps the dose of 0.3 mg/kg caffeine was too low to obtain significant ergogenic effects; consequently, this limitation should be addressed in future studies.

## 5. Conclusions

At the end of this study, it was found that the three ergogenic aids together did not generate a summative effect on the physical performance of young soccer players. However, it is important to analyze individual responses to these specific protocols.

## 6. Practical Applications

Before starting creatine or caffeine supplementation, the following are suggested: (a) adequate supervision, (b) that participants engage in serious/competitive training, (c) that they consume a balanced diet that helps improve performance, (d) that they know the proper use of supplements, and (e) that they do not exceed the recommended doses [16,24]. In parallel, regardless of the method used to trigger PAPE, when planning training loads, coaches and athletes must consider the training objectives and the magnitude of the effect of the different methodologies available [57]. Finally, and above all, they must test individual responses to various stimuli [45].

## 7. Perspective

In light of the results, a creatine supplementation equivalent to 3.0 g/kg/day for 14 days and a single dose of caffeine equivalent to 0.3 mg/kg did not generate adverse effects in the young soccer players in this study. Consequently, these ergogenic aids, taken together, are safe for these athletes. Therefore, future studies could test higher and prolonged doses of creatine and caffeine and evaluate the effect on physical performance in this population.

## Figures and Tables

**Table 1:** Age, anthropometric characteristics, and basal physical performance.

	EG_1_ (*n* = 7)	EG_2_ (*n* = 7)	EG_3_ (*n* = 7)	EG_4_ (*n* = 7)	ANOVA *p*-Value
Age (years)	17.4 ± 1.2	17.1 ± 0.8	17.4 ± 0.9	16.7 ± 0.7	ns
Body weight (kg)	66.9 ± 4.2	69.0 ± 6.4	68.0 ± 5.2	70.2 ± 8.4	ns
Stature (m)	1.71 ± 5.3	1.73 ± 8.4	1.72 ± 6.4	1.73 ± 7.3	ns
BMI (kg/m^2^)	19.4 ± 0.8	19.9 ± 1.3	19.7 ± 0.9	20.1 ± 2.1	ns
Fat mass (kg)	14.3 ± 2.3	12.0 ± 3.9	14.7 ± 1.6	15.7 ± 3.8	ns
1RM (kg)	135.6 ± 13.2	143.3 ± 19.4	132.6 ± 22.3	138.9 ± 22.7	ns
RSA (s)	6.93 ± 0.2	7.03 ± 0.2	6.96 ± 0.1	7.01 ± 0.2	ns

Kg: kilograms; kg/m^2^: kilograms per square meter; ns: not significant; RSA: repeated sprint ability; and s: seconds.

**Table 2:** Mean and SD values of RSA and HBS.

Variable	Treatment	BL	S1	S2	S3
		Mean ± SD	Mean ± SD	Mean ± SD	Mean ± SD
RSA Outcomes					
Best time (s)	EG_1_	6.93 ± 0.29	6.96 ± 0.18	7.05 ± 0.22	7.07 ± 0.17
	EG_2_	7.03 ± 0.21	6.94 ± 0.24	7.05 ± 0.21	6.98 ± 0.18
	EG_3_	6.96 ± 0.16	7.00 ± 0.19	7.02 ± 0.11	7.08 ± 0.11
	EG_4_	7.01 ± 0.22	7.01 ± 0.15	7.08 ± 0.22	7.10 ± 0.29
Mean time (s)	EG_1_	7.09 ± 0.31	7.18 ± 0.22	7.24 ± 0.22	7.30 ± 0.18
	EG_2_	7.28 ± 0.30	7.19 ± 0.24	7.27 ± 0.17	7.22 ± 0.16
	EG_3_	7.22 ± 0.20	7.17 ± 0.16	7.21 ± 0.11	7.34 ± 0.13
	EG_4_	7.27 ± 0.22	7.23 ± 0.20	7.30 ± 0.20	7.34 ± 0.18
Total time (s)	EG_1_	49.64 ± 2.22	50.32 ± 1.55	50.74 ± 1.54	51.10 ± 1.26
	EG_2_	50.99 ± 2.12	50.38 ± 1.71	50.90 ± 1.25	50.58 ± 1.18
	EG_3_	50.56 ± 1.41	50.21 ± 1.14	50.52 ± 0.83	51.39 ± 0.94
	EG_4_	50.93 ± 1.56	50.64 ± 1.45	51.13 ± 1.41	51.41 ± 1.26
Best speed (km/h)	EG_1_	15.55 ± 0.69	15.47 ± 0.41	15.27 ± 0.48	15.21 ± 0.35
	EG_2_	15.31 ± 0.43	15.52 ± 0.55	15.28 ± 0.43	15.42 ± 0.41
	EG_3_	15.47 ± 0.35	15.38 ± 0.41	15.32 ± 0.24	15.21 ± 0.25
	EG_4_	15.35 ± 0.49	15.34 ± 0.32	15.20 ± 0.50	15.18 ± 0.66
Mean speed (km/h)	EG_1_	15.21 ± 0.69	14.98 ± 0.46	14.85 ± 0.44	14.75 ± 0.35
	EG_2_	14.78 ± 0.57	14.98 ± 0.51	14.82 ± 0.38	14.90 ± 0.36
	EG_3_	14.91 ± 0.42	15.01 ± 0.33	14.91 ± 0.26	14.68 ± 0.25
	EG_4_	14.81 ± 0.45	14.88 ± 0.44	14.75 ± 0.43	14.67 ± 0.36
FI (%)	EG_1_	2.28 ± 0.76	3.20 ± 1.25	2.79 ± 1.49	3.17 ± 1.33
	EG_2_	3.53 ± 1.39	3.63 ± 1.45	3.08 ± 0.95	3.53 ± 0.75
	EG_3_	3.69 ± 1.31	2.43 ± 0.96	2.77 ± 1.58	3.68 ± 3.05
	EG_4_	3.72 ± 1.22	3.17 ± 1.56	3.06 ± 1.48	3.46 ± 2.52
HBS outcomes					
Mean velocity (m/s)	EG_1_	0.69 ± 0.04	0.71 ± 0.07	0.71 ± 0.07	0.67 ± 0.09
	EG_2_	0.69 ± 0.02	0.67 ± 0.08	0.67 ± 0.06	0.69 ± 0.07
	EG_3_	0.64 ± 0.07	0.68 ± 0.1	0.66 ± 0.06	0.65 ± 0.07
	EG_4_	0.63 ± 0.05	0.62 ± 0.06	0.63 ± 0.08	0.60 ± 0.12
Power (W)	EG_1_	631.5 ± 53.2	651.0 ± 59.2	651.9 ± 59.4	607.9 ± 78.3
	EG_2_	681.4 ± 74.5	638.7 ± 77.2	655.8 ± 47.3	679.9 ± 56.4
	EG_3_	594.8 ± 147.9	631.3 ± 187.4	614.0 ± 136.9	600.7 ± 139.3
	EG_4_	596.9 ± 64.8	525.3 ± 131.3	585.1 ± 54.9	544.8 ± 80.2

BL: baseline; caffeine: 0.3 mg/kg; creatine: 0.3 g/kg; EG_1_: experimental group 1 (creatine + caffeine + I-sVR); EG_2_: experimental group 2 (creatine + PL_2_ + I-sVR); EG_3_: experimental group 3 (PL_1_ + caffeine + I-sVR); EG_4_: experimental group 4 (PL_1_ + PL_2_ + I-sVR); FI: fatigue index; I-sVR: intra-series variable resistance (1 × 4 at 1.0–1.1 m/s + 1 × 5 at 0.6–0.7 m/s); HBS: half-back squat; km/h: kilometers per hour; m/s: meters per second; PL_1_: maltodextrin (0.3 g/kg); PL_2_: maltodextrin (0.3 mg/kg); RSA: repeated sprint ability; S1: set 1; S2: set 2; S3: set 3; s: seconds; SD: standard deviation; %: percentage; and W: watts.

**Table 3:** ANOVA three-way for RSA and HBS outcomes.

**Variables**	**Time**	Creatine vs. Placebo	Caffeine vs. Placebo	Time × Creatine vs. Placebo	Time × Caffeine vs. Placebo	Creatine vs. Placebo × Caffeine vs. Placebo	Time × Creatine vs. Placebo × Caffeine vs. Placebo
	*p*-Value ${ŋ}_{p}^{2}$	*p*-Value ${ŋ}_{p}^{2}$	*p*-Value ${ŋ}_{p}^{2}$	*p*-Value ${ŋ}_{p}^{2}$	*p*-Value ${ŋ}_{p}^{2}$	*p*-Value ${ŋ}_{p}^{2}$	*p*-Value ${ŋ}_{p}^{2}$
RSA Outcomes							
Best time (s)	*p =* 0.314	*p =* 0.416	*p =* 0.682	*p =* 0.915	*p =* 0.773	*p =* 0.633	*p =* 0.901
	ES = 0.151	ES = 0.027	ES = 0.007	ES = 0.021	ES = 0.050	ES = 0.010	ES = 0.025
Mean time (s)	*p =* 0.225	*p =* 0.318	*p =* 0.294	*p =* 0.855	*p =* 0.613	*p =* 0.873	*p =* 0.772
	ES = 0.179	ES = 0.040	ES = 0.055	ES = 0.030	ES = 0.089	ES = 0.001	ES = 0.055
Total time (s)	*p =* 0.223	*p =* 0.320	*p =* 0.295	*p =* 0.855	*p =* 0.615	*p =* 0.872	*p =* 0.771
	ES = 8.828	ES = 1.961	ES = 2.703	ES = 1.500	ES = 4.379	ES = 0.063	ES = 2.719
Best speed (km/h)	*p =* 0.315	*p =* 0.395	*p =* 0.700	*p =* 0.934	*p =* 0.734	*p =* 0.612	*p =* 0.894
	ES = 0.741	ES = 0.150	ES = 0.032	ES = 0.086	ES = 0.274	ES = 0.055	ES = 0.129
Mean speed (km/h)	*p =* 0.212	*p =* 0.293	*p =* 0.311	*p =* 0.887	*p =* 0.618	*p =* 0.902	*p =* 0.719
	ES = 0.781	ES = 0.188	ES = 0.223	ES = 0.106	ES = 0.383	ES = 0.003	ES = 0.286
FI (%)	*p =* 0.522	*p =* 0.719	*p =* 0.218	*p =* 0.313	*p =* 0.912	*p =* 0.568	*p =* 0.841
	ES = 4.520	ES = 0.258	ES = 4.456	ES = 7.249	ES = 1.505	ES = 0.943	ES = 2.381
HBS outcomes							
Mean velocity (m/s)	*p =* 0.732	*p* < 0.001	*p =* 0.102	*p =* 0.979	*p =* 0.743	*p =* 0.500	*p =* 0.831
	ES = 0.006	ES = 0.066	ES = 0.018	ES < 0.001	ES = 0.008	ES = 0.003	ES = 0.005
Power (W)	*p =* 0.848	*p =* 0.001	*p =* 0.625	*p =* 0.989	*p =* 0.438	*p =* 0.054	*p =* 0.809
	ES = 7748	ES = 11,1706	ES = 2477	ES = 1116	ES = 28,253	ES = 39,951	ES = 9910

ES: effect size; HBS: half-back squat; km/h: kilometers per hour; m/s: meters per second; *p*: *p*-value; RSA: repeated sprint ability %: percentage; W: watts; and ${ŋ}_{p}^{2}$: partial eta-squared.

## Data Availability

Data are contained within the article and Supplementary Materials.

## Supplementary Materials

Supplementary Material Table S1: https://doi.org/10.6084/m9.figshare.25913716; Supplementary Material Table S2: https://doi.org/10.6084/m9.figshare.25917778; Supplementary Material Table S3: https://doi.org/10.6084/m9.figshare.25917784; Supplementary Material Table S4: https://doi.org/10.6084/m9.figshare.25917787; Supplementary Material Table S5: https://doi.org/10.6084/m9.figshare.25917790; Supplementary Material Table S6: https://doi.org/10.6084/m9.figshare.25917817; Supplementary Material Table S7: https://doi.org/10.6084/m9.figshare.25917820; and Supplementary Material Table S8: https://doi.org/10.6084/m9.figshare.25917823.
